# Phenotypic Characterization and Comparative Genomic Analyses of Mycobacteriophage WIVsmall as A New Member Assigned to F1 Subcluster

**DOI:** 10.3390/cimb45080406

**Published:** 2023-08-03

**Authors:** Xinge Guo, Jing Zhang, Yuhan Wang, Fang Zhou, Qiming Li, Tieshan Teng

**Affiliations:** Institute of Biomedical Informatics, School of Basic Medical Sciences, Henan University, Kaifeng 475004, Chinawangyh@henu.edu.cn (Y.W.);

**Keywords:** mycobacteriophage, mycobacterium, antibiotic-resistant bacteria

## Abstract

In this study, we conducted the morphological observation, biological and genomic characterization, evolutionary analysis, comparative genomics description, and proteome identification of a recently isolated mycobacteriophage, WIVsmall. Morphologically, WIVsmall is classified as a member of the Siphoviridae family, characterized by a flexible tail, measuring approximately 212 nm in length. The double-stranded phage genome DNA of WIVsmall spans 53,359 base pairs, and exhibits a G + C content of 61.01%. The genome of WIVsmall comprises 103 protein-coding genes, while no tRNA genes were detected. The genome annotation unveiled the presence of functional gene clusters responsible for mycobacteriophage assembly and maturation, replication, cell lysis, and functional protein synthesis. Based on the analysis of the phylogenetic tree, the genome of WIVsmall was classified as belonging to subgroup F1. A comparative genomics analysis indicated that the WIVsmall genome exhibited the highest similarity to the phage SG4, with a percentage of 64%. The single-step growth curve analysis of WIVsmall revealed a latent period of 120 min, and an outbreak period of 200 min.

## 1. Introduction

**Figure 1:**
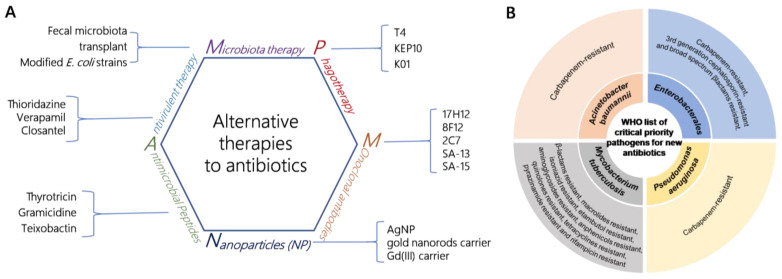
(**A**) Antibiotic replacement therapy strategies; (**B**) WHO list of critical pathogens for the development of new antibiotics.

As a crucial milestone in modern medicine, antibiotics have played a pivotal role in controlling pathogenic infections, and saving innumerable lives, over the past century. However, as bacteria have evolved multiple mechanisms to impede the efficacy of antimicrobial agents, it is imperative that we discover innovative approaches to combating drug-resistant bacterial infections [1]. In fact, there are now numerous alternative therapies available to combat drug resistance, including monoclonal antibodies, and microbiota therapy [2]. However, bacteriophages have emerged as a highly promising alternative to antibiotics (Figure 1A). Compared with antibiotics, bacteriophages offer several significant advantages against bacterial infections, including host specificity, the absence of cross immunogenicity with antibiotics [3], abundance in nature [4], and self-replication [5], improving the speed and quality of treatment. Thus far, bacteriophages have proven effective in treating various clinical cases of antibiotic-resistant bacterial infections. For example, a 63-year-old patient diagnosed with a urinary tract infection caused by *K. pneumoniae* was successfully treated through a phage cocktail therapy, comprising six phages in combination with anti-infective drugs, resulting in clinical cure [6]. Additionally, bacteriophage therapy has demonstrated efficacy in treating infections caused by *S. aureus*, *E. coli*, *A. bambini*, and *M. abscessus* [7,8]. Although the clinical application of mycobacteriophages for treating *M. abscessus* infections has been successful, their widespread implementation is challenging, due to their limited efficacy against other clinically isolated *M. abscessus* strains. The clinical application of phage therapy may be perceived as a form of personalized therapy, which may limit its accessibility to those with the financial means.

The World Health Organization (WHO) released a global priority list of pathogens in 2017 [9]. The pathogens were categorized as critical-, high-, and medium-priority bacteria, based on the urgency of developing new antibiotics to combat them. *Mycobacterium tuberculosis* has been identified as one of the high-priority bacteria. *M. tuberculosis* is the causative agent of pulmonary tuberculosis (TB), a disease that poses a significant threat to human health. It is estimated that approximately one quarter of the world’s population is infected with *M. tuberculosis*, with over 1.4 million deaths attributed to tuberculosis each year [10]. The emergence of drug-resistant strains of *M. tuberculosis* poses a significant obstacle to the effective control of tuberculosis [11]. Based on the 2022 World Health Organization (WHO) report on tuberculosis (TB), the proportion of multi-drug resistant cases was estimated as being 20% in 2021, and almost 43% of MDR-TB cases were treated between 2018 and 2021 [12]. Mycobacteriophages, either in isolation or as a cocktail, or as part of a synergistic therapy with antibiotics, have potential in the treatment of mycobacterial infections [13]. It is reported that 20 patients with antibiotic-refractory mycobacterial infections received treatment with a mycobacteriophage cocktail [14]. Favorable clinical or microbiological responses were observed in 11 of these patients. Additionally, Jessica S. Little and her team reported a case of a 56-year-old patient with a refractory disseminated cutaneous *Mycobacterium chelonae* infection, who received mycobacteriophage therapy, and experienced a significant improvement in their skin lesions [15]. Mycobacteriophages can also be utilized as a tool for engineering shuttle plasmids to detect drug-resistant *M. tuberculosis* strains, including DS6A and TM4-based shuttle plasmids [13,16].

## 2. Materials and Methods

### 2.1. Phage Isolation and Preparation

*M. smegmatis* mc^2^155 served as the host bacterium for the isolation of the mycobacteriophages. The isolation of WIVsmall was carried out in accordance with a previously described methodology [17]. For the phage isolation, 10 g of soil sample was mixed with 10–15 mL of buffer (50 mM Tris-HCl, pH 7.5, 150 mM NaCl, 10 mM MgSO_4_, and 2 mM CaCl_2_), and 1 mL of *M. smegmatis* mc^2^155, in a 50 mL Erlenmeyer flask. The mixture was incubated at 37 °C for 24 h. The supernatant was filtered using a 0.22-μm membrane filter, to eliminate residual bacteria and phytoplankton. The conventional double-layer agar technique and spot assay were employed to isolate individual phages. The process of single plaque-picking was repeated thrice. The phage lysates were subsequently stored at 4 °C for further experimentation

### 2.2. DNA Extraction

Genomic DNA was extracted from the purified phage using SDS-proteinase K protocols [18]. The structural protein of the phage WIVsmall was digested, using a combined reagent consisting of 50 mg of protease K (Invitrogen, Shanghai, China), 20 mM EDTA, and 1% SDS, for 4 h at 56 °C. The phage DNA was extracted using the conventional phenol–chloroform method, with an equal volume of phenol/chloroform/isoamyl alcohol (25:24:1, *v/v*). The aforementioned step was repeated, and an equal volume of chloroform was employed to further refine the supernatant. The combined reagent, consisting of one volume of 2-propanol, and 0.4 volumes of 3M sodium acetate (pH 4.6), was added to the supernatant. The resulting mixture was then centrifuged at 7000× *g* for 10 min at 4 °C, to precipitate the phage DNA.

### 2.3. Sequence Data and Phylogenetic Analysis

The complete genome was sequenced, using 454 technology, with the GS Junior 454 system platform (Roche Diagnostics, Indianapolis, IN, USA), yielding 145-fold coverage of the phage genome. The 15 available nucleotide sequences of the mycobacteriophage genomes in the F cluster were retrieved from the NCBI nucleotide database. Based on the phage coding sequence, a phylogenetic tree was drawn, using the neighbor linkage (NJ) method. The bootstrap value was 1000 replicates on MEGA X [19].

### 2.4. Optimal Multiplicity of Infection (MOI)

*M. smegmatis* mc^2^155 was cultured to the exponential phase, and subsequently washed thrice with 7H9 medium, to eliminate Tween-80. The bacterial pellets were resuspended in 7H9 medium, and the cell concentration was adjusted to 10^6^ colony-forming units per milliliter (CFU/mL). The phages were mixed with *M. smegmatis* mc^2^155 at varying dilutions, including the ratios of 10:1, 1:1, 1:10, 1:100, and 1:1000. The aforementioned mixtures were incubated at 37 °C, with agitation for 12 h, at 177 revolutions per minute. Subsequently, the 1 mL mixture was centrifuged at 10,000× *g* for 20 min, to remove the precipitated bacteria. The resulting supernatant was then filtered through a 0.22 µm pore-size membrane filter. The phage filtrate was detected through the employment of the gradient dilution method, and a double-layer agar (DLA) assay, as previously described [20]. The dilution that generated the highest phage titer was considered as the optimal MOI.

### 2.5. Single-Step Growth Experiment

To determine the single-step growth curve, 2 mL of *M. smegmatis* mc^2^155, with a titer of 10^6^ CFU/mL, was mixed with 2 mL of the WIVsmall phage solution, at the optimal MOI, and incubated at 37 °C for 25 min. The mixture was centrifuged at 12,500× *g* for 2 min, to remove unabsorbed phage particles. The precipitated *M. smegmatis* mc^2^155 was suspended in mycobacteriophage buffer, and incubated at 37 °C and 177 rpm. The supernatant was collected to detect the phage titer, using the DLA method, every 30 min. The experiment was repeated three times, and a single-step growth curve was plotted.

### 2.6. Stability of the Phage under Various Conditions

The stability of the phage under several conditions was detected, as previously described, with some modifications [21]. To determine the phage stability at different temperatures, phage suspensions with a titer of 10^9^ PFU/mL were incubated at 4 °C, 10 °C, 20 °C, 30 °C, 40 °C, 50 °C, 60 °C, 70 °C, 80 °C, and 90 °C for 2 h. To evaluate the ultraviolet (UV) stability of the phage WIVsmall, phage preparations with a titer of 10^9^ PFU/mL were illuminated using a UV lamp (365 nm, 18µW/cm^2^) for 20, 40, 60, 80, 100, and 120 min. Subsequently, the phage titers were determined, using the DLA method. Three parallel experiments were conducted. To assess the pH stability of the phage WIVsmall, 200 µL of phage lysates was diluted in 1.8 mL of MP buffers, at various pH values ranging from 3–12, and incubated at 37 °C for 2 h.

### 2.7. Genomic Bioinformatics Analysis

Softberry (http://linux1.softberry.com/berry.phtmltopic=virus0&group=programs&subgroup=gfindv, accessed on 20 May 2023) and GeneMarkTM (http://exon.gatech.edu/GeneMark/, accessed on 20 May 2023) were used to predict the ORFs of the WIVsmall genome [22]. The starting codons were selected as ATG, TTG, and GTG. Sequences with bases less than 150 bp, and ORFs without SD sequences were manually deleted. The active domains and isoelectric points of ORFs with protein functions were predicted using Pfam (http://pfam.sanger.ac.uk/, accessed on 20 May 2023) [23] and Isoelectric Point Finder (http://greengene.uml.edu/programs/Find MW.html, accessed on 20 May 2023). tRNAscan-SE (http://lowe lab.ucsc.edu/tRNAscan-SE/, accessed on 20 May 2023) [24] and Aragorn (http://mbio-serv2.Mbioekol.lu.se/ARAGORN/, accessed on 20 May 2023) [25] were used to search for the tRNA-encoding genes in the genome. BLASTn (http://blast.ncbi.nlm.nih.gov/Blast.cgi, accessed on 20 May 2023) and FASTA (http://www.ebi.ac.uk/Tools/fasta33/index.html, accessed on 20 May 2023) were used to perform the DNA sequence alignment. Multiple genome alignments were performed using the software easyfig. CGview (http://wishart.biology.ualberta.ca/cgview/, accessed on 20 May 2023) [26] and GenomeVx (http://wolfe.ucd.ie/GenomeVx/, accessed on 20 May 2023) were used in the visual analysis of the genomic maps. MEGA-X was used to perform neighbor-joining evolutionary tree analysis via 1000 bootstrap replications [19]. DNAPlotter was used to detect the classification of the bacteriophage clusters [27].

### 2.8. Identification of Phage Structural Proteins

Polyethylene glycol 8000 powder was added to the bacteriophage solution to achieve a final concentration of 20%, followed by overnight precipitation at 4 °C. The phage precipitate was collected via centrifugation at 12,000 rpm for 30 min, and then re-suspended in a dithiothreitol (DTT) solution. The lysate was placed on ice, and subjected to pulse ultrasound treatment. The ultrasonically treated samples were electrophoresed ona 12% SDS-PAGE, and subsequently stained with Coomassie Brilliant Blue R250. The protein bands were excised, digested with trypsin, and subsequently analyzed via mass spectrometry.

## 3. Results

### 3.1. Phage Isolation and Morphology Analysis of Plaque

**Figure 2:**
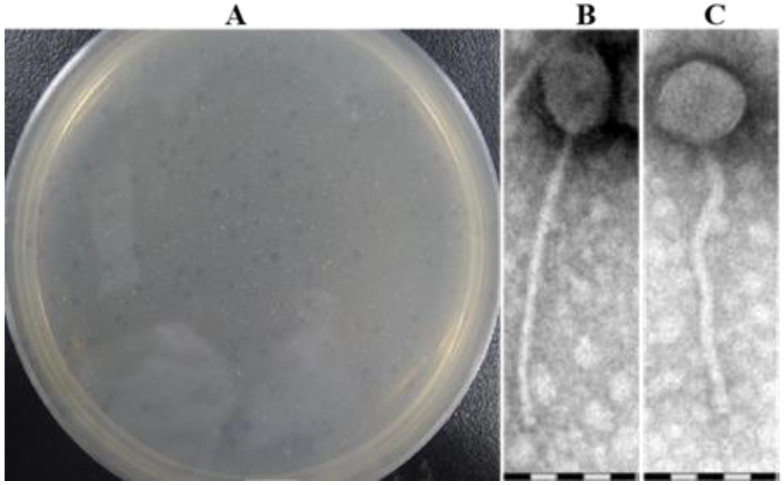
(**A**) Plaque morphology of WIVsmall; (**B**,**C**) TEM showed that the phage WIVsmall had an equal-length head attached to a retractable tail.

The mycobacteriophage WIVsmall was isolated from soil samples collected in Henan Province, China, using *M. smegmatis* mc^2^155 as the host organism. The plaques of WIVsmall manifested as pinpricks accompanied by a slight turbidity on an *M. smegmatis* mc^2^155 lawn (Figure 2A), indicating a low frequency of lysogeny [28]. This result is consistent with the plaque formation characteristics of temperate mycobacteriophages [29]. The TEM analysis of purified phage particles revealed that the phage WIVsmall, a member of the Siphoviridae family, comprised an isometric head with a diameter of approximately 72 nm, and a long, flexible tail with a diameter of approximately 212 nm (Figure 2B,C).

### 3.2. Optimal Multiplicity of Infection (MOI) and One-Step Growth Curve

**Figure 3:**
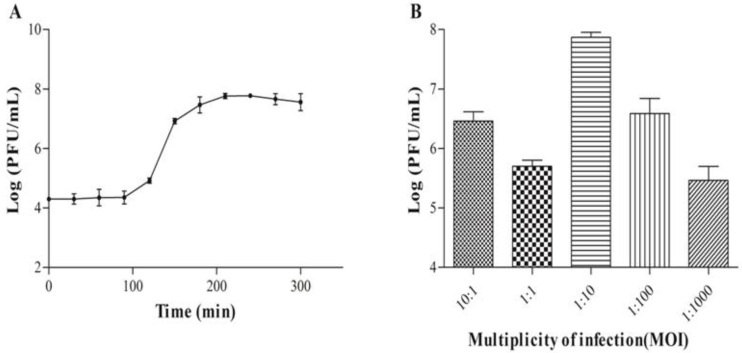
Single-step growth experiment of the mycobacteriophage WIVsmall (**A**); and analysis of the optimal MOI (**B**).

A familiarity with the growth characteristics of bacteriophages is a prerequisite to investigating their life cycle. We conducted an analysis of the optimal multiplicity of infection, and the one-step growth curve of the phage WIVsmall. To ascertain the optimal multiplicity of infection (MOI), the phage WIVsmall was incubated with its host, *M. smegmatis* mc^2^155, under varying MOI conditions. The MOI of 0.1 could yield the highest titer of the progeny phage (Figure 3B), and could be considered the optimal MOI. The single-step growth curve analysis exhibited that WIVsmall had a latent period of approximately 120 min, and a burst size of approximately 12.8 PFU per infected cell after 200 min of incubation at 37 °C (Figure 3A). Like other temperate phages, the extended latency period and limited burst size of WIVsmall can likely be attributed to the low activity of its DNA polymerase [30].

### 3.3. General Genome Analysis

**Figure 4:**
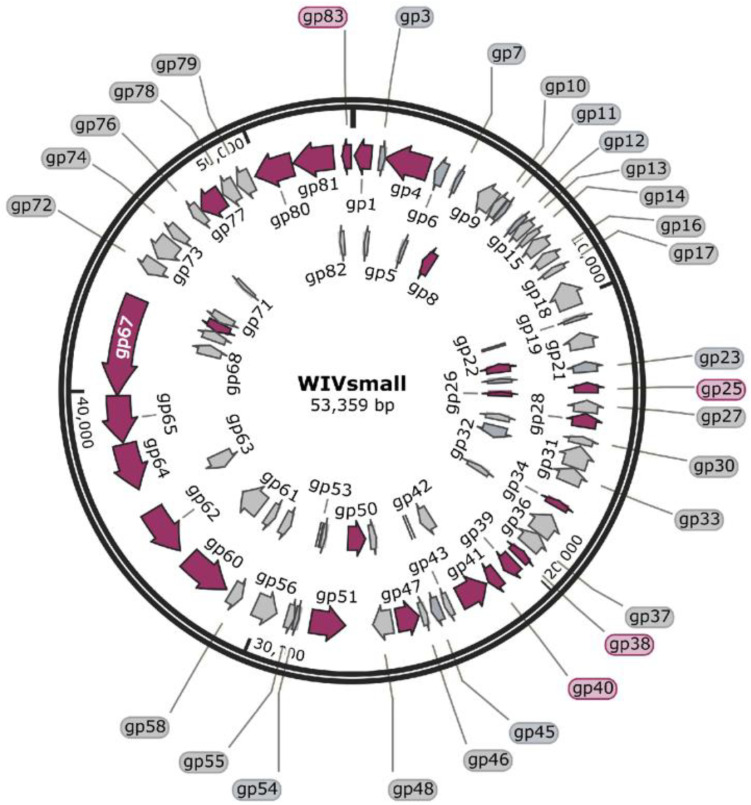
Map of the phage WIVsmall genome.

The genomic sequence has been deposited in the NCBI database, under the accession number GenBank: KC736071.1. The genome size of WIVsmall was 53,359 bases, with a G + C content of 61.01%, which is comparable to that of its host mycobacteria, and other sequenced mycobacteriophages. The genome harbored 103 open reading frames (ORFs), spanning from 99 bp to 3639 bp, and no putative genes encoding tRNA or tmRNA were identified. WIVsmall demonstrated a condensed genome organization (Figure 4), and the 3′-terminal genome’s sticky end sequence was CGGACGGCGC. Based on sequence comparisons within the Actinobacteriophage Database (phaseDB.org), all the ORFs exhibited sequence homology with other entries in the database. Among them,48 ORFs have been functionally characterized, while the remaining 55 ORFs exhibit homology to proteins that have not yet been characterized. There are 82 genes exhibiting mosaic structures in the genome of WIVsmall, suggesting that these genes may have undergone horizontal gene transfer, which is comparable to other F1 cluster mycobacteriophages. Compared to the majority of the phages in the F1 cluster, the genome of WIVsmall exhibits the conspicuous absence of eight coding genes, three of which encode proteins with well-defined functions, namely carboxypeptidase, DNA methylase, and endonuclease. Among the three functional proteins, endonuclease was found to have multiple homologs with an identical function in the genome of the phage WIVsmall, while no homologs with an identical function were identified for the remaining two proteins. However, other F1 cluster phages, including Cerasum and BigPhil, with genome lengths comparable to WIVsmall, were also found to lack coding genes for carboxypeptidase and methylase. Table 1 presents the anticipated dimensions, location, transcriptional alignment, and the nearest phage protein analogue.

### 3.4. Putative Functions of the Predicted ORFs

The bioinformatics analysis revealed that the WIVsmall genome presented a functional mosaic structure. The WIVsmall genome can be partitioned into four distinct functional modules: phage assembly and maturation, replication, cell lysis, and functional proteins. The genes associated with phage assembly and maturation are located on the left arm of the genome, comprising five ORFs. ORF80 and ORF79 exhibit a significant sequence similarity with the large and small subunits of the terminal enzymes, respectively. These enzymes are accountable for the assembly of the phage DNA into the capsid. Three site-specific DNA endonucleases, encoded by ORF67, ORF48, and ORF23, are implicated in the horizontal transfer of phage genes.

The lysis module of WIVsmall comprises three contiguous open reading frames (ORF49, ORF50, and ORF51). The protein encoded by ORF50 exhibits a significant similarity to LysinA, and demonstrates catalytic activity toward the peptidoglycan layer of mycobacterial cell walls. ORF51 encodes the LysinB protein, which functions as a mycolylarabinogalactan hydrolase enzyme, capable of cleaving the linkage of the PG–AG polymer and, thereby, facilitating the detachment of the unique mycolic acid layer of the mycobacterial cell wall [31]. The deletion of the *lysinB* gene has been shown to decrease both the plaque and burst sizes [32]. ORF49 encodes the drilling protein holin, which facilitates the formation of pores in the cytoplasmic membrane, thereby enabling the release of LysinA and LysinB from the cytoplasm to the target cell wall [33].

The DNA replication module comprises ORFs encoding proteins involved in DNA replication and transcription. ORF7 encodes a DNA polymerase, whereas ORF38 and ORF25 exhibit significant sequence homology with transcriptional regulators belonging to the Xre family. ORF28 and ORF26 encode putative transcriptional regulatory proteins of the WhiB family, which recognize promoter regions, and regulate gene expression in the phage WIVsmall [34]. In general, primer enzymes and helicases are essential to the initiation of DNA replication. However, the absence of primases and helicases in the genome of the phage WIVsmall suggests its dependence on the host for genome replication, repair, and transcription. A putative integrase (ORF41) was also predicted, which can determine whether phages undergo lysogenic or lytic cycles.

The morphology module encompasses genes that encode the structural proteins of WIVsmall. Three adjacent ORFs, namely ORF77, ORF78, and ORF79, were identified as encoding the major capsid protein, head-scaffolding protein, and head-maturation protease, respectively. These proteins are believed to play crucial roles in stabilizing the condensed form of DNA, and facilitating head development. ORF63 and ORF44 encode large and small tail proteins, respectively. The ORFs involved in the tail structure formation comprise minor tail proteins, major tail proteins, and tail-assembly chaperones.ORF67 encodes a tape-measure protein that precisely measures the length of the bacteriophage tail.ORF78 encodes a portal protein capable of facilitating the transfer of the phage DNA into host cells through the formation of a channel.

### 3.5. Phylogenetic Relationships

**Figure 5:**
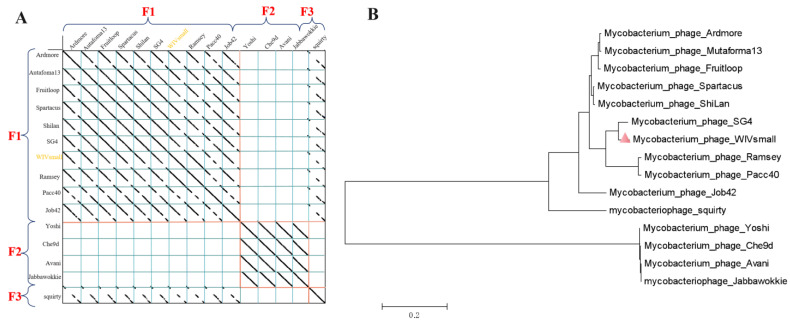
(**A**) Dot plot of parts of the F cluster mycobacteriophage genomes, displayed using Gepard. All genome sequences are connected into one sequence, so that related genomes are adjacent to each other. (**B**) Phylogenetic tree of the mycobacteriophage WIVsmall isolates. The neighbor-joining tree is based on the amino acid sequence alignment of the available sequences in GenBank and the mycobacteriophage database.

The phage genome was partitioned into clusters and subclusters, using dot mapping. As per Hatfull’s work [35,36], two genomes can be assigned to the same cluster if their sequences in the dot plot exhibit a similarity higher than 50%. Moreover, a cluster can be partitioned into subclusters if the relationships within the cluster are heterogeneous. All the genomes within the same subcluster typically exhibit a greater sequence similarity. The dot-plot results for WIVsmall and other F1 cluster phages indicate that WIVsmall is likely a member of the F1 subcluster (Figure 5A). To investigate the evolutionary position of WIVsmall within the mycobacteriophage family, we conducted the phylogenetic analysis of the tape-measure protein (ORF67), which is the longest gene in the mycobacteriophage genomes. The tape-measure-protein-encoding gene is highly conserved, and serves as a typical phylogenetic marker for mycobacteriophages [37]. The amino acid sequences of the tape-measure protein from WIVsmall, and 14 closely related mycobacteriophages (F1cluster) were aligned. A phylogenetic tree was then constructed, using MEGA X software. WIVsmall was found to be most closely related to SG4, which belongs to the F1 subcluster of mycobacteriophages (Figure 5B).

### 3.6. Comparative Genomics Analysis

**Figure 6:**
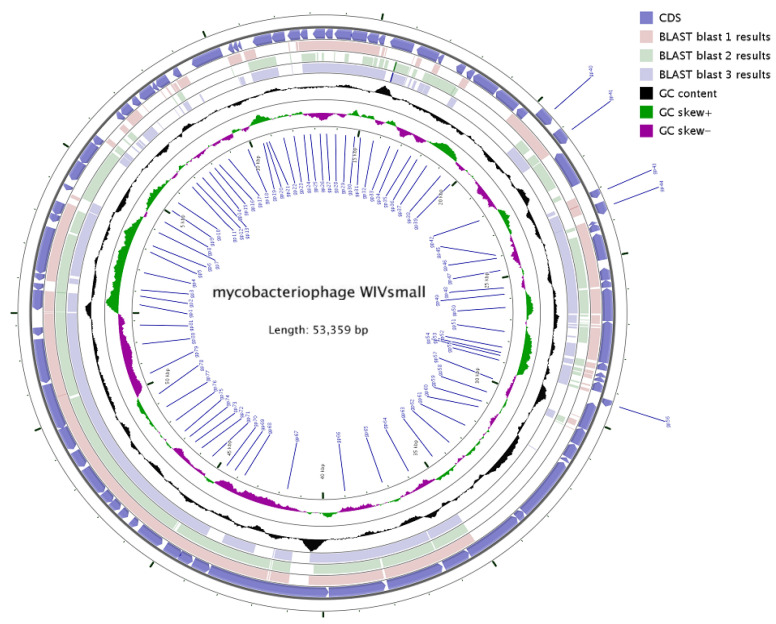
Genetic and physical maps were prepared using CGview. Blast 1–3 revealed the Genomic sequence similarity of WIVsmall with SG4, Bobi, and Boomer, respectively. Three annular trajectories were described (from inside to outside): GC tilt ([G − C]/[G + C]), with inward peaks indicating a larger proportion of G; GC content (the inner peak is lower than the average GC content); and ORFs and transcription direction.

Based on BLASTn analysis, 15 phages belonging to the mycobacteriophage F cluster exhibiteda significant similarity to the WIVsmall genome. Among them, the mycobacteriophage SG4 exhibited the highest similarity, 64%, with the genome (Table 2). Meanwhile, a comparative genome analysis was conducted among the phages WIVsmall, SG4, Bobi, and Boomer, using CGview. As depicted in Figure 6, the genes implicated in phage structure and assembly exhibited a high degree of similarity among the three genomes. By conducting a BLASTp comparison against the Actinobacteriophage Database (phaseDB.org), we identified three proteins (Gp58, Gp59, and Gp61) in the WIVsmall genome that exhibited no similarities with the mycobacteriophage belonging to cluster F1 (Table 3). Among them, Gp58 exhibited homology with proteins found in non-mycobacteriophages, including those of the Tsukamurella phage, Gordonia phage, and Rhodococcus phage. Notably, all of the host bacteria for these phages, as well as mycobacteria, belong to the Actinomycetales taxonomic order. Gp59 exhibiteda similarity to proteins found in mycobacteriophages, including IdentityCrisis, Shweta, Ruthiejr, Willsammy, and Taquito, none of which are classified as members of the F1 cluster. Gp61 displayed homology with the protein found in the phage Moosehead, which was obtained utilizing *Gordonia terra* 3612 as the host. However, Gp61 also exhibited a significant homology with proteins originating from mycobacteriophages, including Wilder, MkaliMitinis3, LilDestination, and Lewan. Notably, none of these mycobacteriophages are affiliated with the F1 cluster. After performing BLASTpanalysis against the Actinobacteriophage Database, we found that the remaining 100 proteins exhibited a homology with other mycobacteriophages belonging to the F1 cluster. However, the coverage and identity values of two proteins, Gp34.1 and Gp60, were below 40%, casting doubt on the functional analysis results obtained through sequence alignment.

### 3.7. Biological Characteristics of the WIVsmall Phage

**Figure 7:**
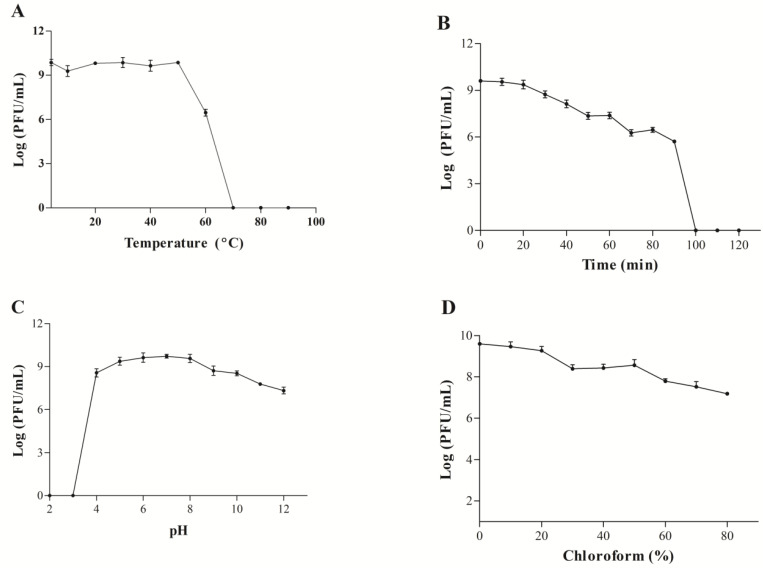
(**A**) Thermal stability test; (**B**) UV stability test; (**C**) pH stability test; (**D**) and chloroform sensitivity.

When cultured at temperatures of 50 °C or lower, the bacteriophage WIVsmall remained infectious to *M. smegmatis* mc^2^155, demonstrating an exceptional thermal stability. However, the phage titer gradually decreased in the water bath above 50 °C. Consistently, the titer of the phage WIVsmall remained undetectable at temperatures exceeding 70 °C (Figure 7A). The bacteriophage WIVsmall exhibited stability within the pH range of 4 to 11, over a duration of 1 h. However, under the acidic conditions of pH 2 to 3, the survival rate of the phages was negligible (Figure 7C). Furthermore, chloroform did not significantly impact WIVsmall’s infectivity toward *M. smegmatis* mc^2^155, indicating that WIVsmall is a lipid-free bacteriophage (Figure 7D).

### 3.8. Mass-Spectrometric Identification of Phage Proteins

The LC-MS/MS analysis identified 10 out of the 103 predicted ORF expression products. Among them, eight were phage structural proteins, including three types of phage minor tail protein, a putative structural protein, a tail-length tape measure protein, a major tail protein, a major capsid subunit, and a putative portal protein (Table 4). The absence of a significantly similar sequence in the database led to the classification of two additional proteins, ORF61 and ORF66, as uncharacterized proteins. However, the LC-MS/MS analysis failed to detect other predictions as hypothetical and functional proteins. This could be attributed to the low expression levels of the aforementioned proteins, which are beyond the detection limit of LC-MS/MS analysis. Additionally, these structural proteins exhibit a higher degree of similarity to mycobacteriophages possessing comparable genome sizes, such as SG4, Ramsey, Squirty, and Job42, than to other phages.

## 4. Discussion

In recent years, the irrational use of antibiotics has resulted in the continuous emergence of drug-resistant bacteria, and even “superbugs”, ushering in a “post-antibiotic era”, where in effective antibiotics are no longer available to combat infections in humans. *M. tuberculosis* is widely recognized as one of the most formidable drug-resistant bacterial pathogens in clinical settings. Multidrug-resistant tuberculosis (MDR-TB) and extensively drug-resistant tuberculosis (XDR-TB) constitute 20% of all cases of primary tuberculosis [38]. Several studies have tackled this challenge by exploring the immense potential of mycobacteriophages in TB detection and treatment [39]. In this study, we have identified and sequenced a novel phage, WIVsmall, that infects *M. smegmatis* mc^2^155. Dot-plot and phylogenetic analyses indicate that WIVsmall belongs to the F1 subcluster, as a novel member. According to the Actinobacteriophage Database (PhageDB.org), the genome length of the phages belonging to the F1 cluster ranges from 52,141 bp to 61,164 bp, while the number of genes encoded by the phages in this cluster ranges from 88 to 113. The total length of the WIVsmall genome is 53,359 bp, comprising 103 coding genes, which falls within the range of the phage F1 cluster. Comparative genomic analysis revealed that WIVsmall shares a maximum sequence similarity of only 64% with other mycobacteriophages. WIVsmall exhibited remarkable stability across diverse conditions, encompassing pH and temperature, which suggests its potential suitability in clinical settings. These findings provide new insights into the diversity and evolution of mycobacteriophages, and highlight the potential of WIVsmall as a model system for studying phage–host interactions. Recently, the focus of engineered phage development has primarily been on phages targeting *S. aureus* [40], *E. coli* [41], and *P. aeruginosa* [42], with comparatively fewer studies on engineered mycobacteriophages. Hence, it is imperative to delve deeper into bioengineering research concerning mycobacteriophages in this context.

## Figures and Tables

**Table 1:** Predicted molecular functions of the gene products of the phage WIVsmall.

ORF Number	Start and Stop Position	Strand	Length (bp)	MW (kDa)	Molecular Function	E-Value	Coverage (%)	Identity (%)
1	83–697	−	615	22.83	Glucosyltransferase	2E-142	100	95.59
2	694–918	−	225	8.08	Hypothetical protein	2E-43	100	95.59
3	989–1201	−	213	7.78	Hypothetical protein	2E-42	100	97.14
4	1204–2823	−	1620	61.18	Glycosyltransferase	0.00	100	93.70
5	2820–3029	−	210	7.46	Hypothetical protein	7E-43	100	100
6	3059–3460	−	402	14.36	Hypothetical protein	4E-89	100	99.25
6.1	3457–3984	−	528	20.44	Hypothetical protein	3E-69	71	86.40
6.2	3702–3839	−	138	4.86	Hypothetical protein	1E-24	100	100
7	3866–4030	−	165	6.07	GIY-YIG nuclease	8E-25	90	95.92
7.1	3936–4181	+	246	8.75	Hypothetical protein	1E-27	77	95
8	4217–4819	−	603	22.39	HtrLYibB protein	2E-143	100	98.50
9	4816–5496	−	681	25.77	Hypothetical protein	2E-162	100	100
10	5499–5795	−	297	11.10	Hypothetical protein	3E-53	84	100
11	5832–6020	+	189	6.66	Hypothetical protein	2E-38	100	100
11.1	6167–6469	−	303	10.65	Hypothetical protein	5E-21	67	80.36
12	6531–6716	−	186	7.12	Hypothetical protein	9E-36	100	98.36
13	6719–7075	−	357	13.68	Mobile element MPME	2E-79	96	99.12
14	7158–7385	−	228	8.38	Hypothetical protein	9E-43	97	95.89
15	7444–7932	−	489	17.83	Hypothetical protein	2E-113	100	99.38
15.1	7929–8066	−	138	4.71	Hypothetical protein	8E-22	100	97.78
16	8063–8470	−	408	14.38	Hypothetical protein	1E-37	95	68.09
16.1	8393–8683	+	291	10.59	Hypothetical protein	3E-40	85	91
16.2	8467–8661	−	195	7.07	Hypothetical protein	1E-38	100	98.44
17	8661–8885	−	225	8.37	Hypothetical protein	3E-40	100	93.24
17.1	9041–9304	−	264	9.31	Hypothetical protein	1E-52	100	95.40
18	9301–10,263	−	963	34.65	Replication initiation protein	0.0	100	98.44
18.1	10,474–10,716	−	243	9.25	Hypothetical protein	4E-42	100	86.25
19	10,632–10,778	−	147	5.40	Hypothetical protein	5E-27	100	100
20	10,778–10,882	−	105	3.79	Hypothetical protein	2E-14	100	97.06
20.1	10,879–11,241	−	363	13.72	HNH endonuclease	6E-83	100	98.33
21	11,238–11,816	−	579	20.60	Single strand annealing protein	3E-135	100	99.48
22	11,813–12,295	−	483	18.13	HNH endonuclease	5E-113	100	97.50
23	12,292–12,666	−	375	13.94	DNA methyltransferase	2E-67	100	89.68
24	12,663–12,893	−	231	8.26	Hypothetical protein	3E-43	100	97.37
25	13,048–13,413	−	366	13.39	DNA binding protein	3E-83	100	99.17
26	13,410–13,643	−	234	8.57	WhiB transcriptional factor	3E-44	94	98.63
27	13,692–14,129	−	438	16.57	Hypothetical protein	2E-98	100	100
28	14,174–14,668	−	495	18.60	WhiB transcriptional factor	6E-117	100	98.78
29	14,668–15,015	−	348	12.35	Hypothetical protein	3E-62	99	79.82
30	15,012–15,290	−	279	10.67	Hypothetical protein	7E-56	100	93.48
31	15,369–16,169	−	801	27.87	Hypothetical protein	2E-109	100	76.69
32	15,379–16,041	+	663	22.30	Hypothetical protein	1E-139	100	100
33	16,199–16,765	−	567	20.32	Hypothetical protein	5E-28	100	56.12
33.1	16,873–17,031	−	159	6.10	Hypothetical protein	2E-27	100	100
33.2	17,058–17,489	−	432	16.18	HNH endonuclease	9E-91	100	78.57
34	17,489–17,746	−	258	9.82	Excisionase	1E-51	100	96.51
34.1	17,727–18,215	−	99	5.87	Hypothetical protein	2E-11	100	96.88
34.2	17,743–17,841	−	324	11.63	Hypothetical protein	1E-42	66	98.59
35	17,838–18,161	−	324	11.63	Hypothetical protein	1E-42	66	98.59
36	18,076–18,891	−	816	30.12	Hypothetical protein	2E-26	95	32
37	18,907–19,506	−	600	21.83	Hypothetical protein	2E-85	98	67.30
38	19,729–20,034	−	306	11.50	Transcriptional repressor	2E-67	100	99.01
39	20,218–20,721	+	504	18.73	Transcriptional repressor	3E-112	100	97.60
40	20,984–21,430	+	447	15.55	Pin protein	2E-83	100	91.33
41	21,522–22,640	−	1119	41.52	Integrase	0.0	100	99.73
42	21,960–22,634	+	675	22.65	Hypothetical protein	2E-148	100	100
43	23,014–23,250	+	237	8.49	Hypothetical protein	5E-22	94	62.67
44	23,250–23,417	+	168	8.49	Hypothetical protein	6E-31	100	98.18
45	23,399–23,758	−	360	13.37	Hypothetical protein	2E-77	94	98.23
46	23,928–24,161	+	234	8.39	Hypothetical protein	1E-46	100	98.70
47	24,234–25,055	−	822	31.03	DNA polymerase exonuclease subunit	0.0	100	99.63
47.1	25,042–25,302	−	261	9.92	Hypothetical protein	4E-56	100	100
48	25,224–25,931	−	708	25.33	Hypothetical protein	8E-169	100	100
49	25,299–25,673	−	375	14.21	Minor tail protein	2E-80	100	99.19
49.1	25,670–25,903	−	234	7.78	Holin	7E-43	100	98.70
50	25,920–26,921	−	1002	36.63	Endolysin	0.0	100	98.80
51	26,921–28,198	−	1278	47.42	Endolysin	0.0	100	95.29
52	28,195–28,428	−	234	8.29	Hypothetical protein	2E-41	100	98.70
53	28,499–28,639	−	141	4.94	Hypothetical protein	1E-22	100	95.65
54	28,636–28,785	−	150	5.38	Hypothetical protein	7E-21	95	85.11
55	28,792–29,088	−	297	10.31	Hypothetical protein	4E-41	100	84.69
55.1	29,232–29,444	+	213	8.16	Hypothetical protein	4E-36	100	92.86
56	29,452–30,291	−	840	26.26	Minor tail protein	2E-98	100	90.32
57	30,288–30,815	−	528	17.50	Minor tail protein	2E-50	84	61.49
58	30,830–31,282	−	453	15.63	Hypothetical protein	7E-46	100	62.00
59	31,299–31,745	+	447	15.54	Hypothetical protein	7E-08	32	56.00
60	31,468–33,264	−	1797	59.21	Hypothetical protein	Minor tail protein	7E-50	38
61	32,291–33,682	−	1392	45.56	Minor tail protein	7E-07	67	25
61.1	33,395–33,610	−	216	8.00	Hypothetical protein	6E-16	100	52.44
62	33,660–35,447	−	1788	63.70	Minor tail protein	6E-94	63	49.00
63	35,444–36,289	−	846	28.88	Minor tail protein	0.0	99	98.21
64	36,332–38,065	+	1734	64.58	Minor tail protein	0.0	98	98.77
65	38,124–39,833	−	1710	63.44	Putative structural protein	0.0	100	98.95
66	39,819–42,185	+	2367	88.37	Hypothetical protein	0.0	100	100
67	39,834–43,472	+	3639	122.34	Taillength tape measure protein	0.0	100	98.51
68	42,040–42,633	−	594	20.41	Hypothetical protein	7E-136	100	100
69	42,931–43,464	−	534	18.59	Hypothetical protein	1E-113	100	100
70	43,472–44,011	−	540	20.12	HNH endonuclease	1E-129	100	98.88
71	44,035–44,538	−	504	19.21	Tail assembly chaperone	4E-114	100	98.20
72	44,420–44,971	−	552	20.50	Tail assembly chaperone	2E-129	100	99.45
73	45,090–45,899	−	810	29.78	Major tail protein	0.0	100	99.26
74	46,008–46,412	−	405	14.70	Head–tail adaptor	6E-89	100	99.25
75	46,402–46,626	−	225	7.83	Head–tail connector protein	8E-46	100	98.65
75.1	46,735–47,064	−	330	11.74	Head–tail adaptor	3E-70	100	98.17
76	47,061–47,432	−	372	13.21	Head–tail adaptor Ad1	5E-81	100	99.19
77	47,445–48,353	−	909	31.71	Major capsid subunit	5E-162	99	74.67
78	48,416–48,952	−	537	19.55	Head scaffolding protein	8E-122	100	99.44
79	49,031–49,582	−	552	20.14	Head maturation protease	1E-131	100	98.91
80	49,740–51,161	−	1422	51.43	Portal protein	0.0	100	99.15
81	51,202–52,689	−	1488	53.90	Terminase	0.0	100	99.60
82	52,661–52,915	−	255	9.36	Terminase small subunit	4E-51	100	98.81
83	52,988–53,320	−	333	12.13	HNH endonuclease	3E-73	100	99.09
83.1	53,146–53,358	−	213	7.59	Hypothetical protein	9E-36	95	98.51

“+” respresent the sense strand; “−” respresent the nonsense strand.

**Table 2:** Summary of similar genomic sequences with the phage WIVsmall.

Phage Name	Query Cover	Identity	Accession Number	Genome Size (bp)
SG4	64%	95.74%	NC_026593.1	59,419
Ramsey	63%	96%	NC_011289.1	58,578
Coco12	62%	95.34%	NC_051644.1	57,693
Job42	62%	97.47%	NC_021538.1	59,626
BuzzLyseyear	56%	88.73%	NC_023699.1	59,016
ShiLan	48%	93.36%	NC_041988.1	59,794
Pacc40	47%	93.53%	NC_011287.1	58,554
Squirty	37%	95%	NC_026588.1	60,285
Jabbawokkie	22%	95.03%	NC_022069.1	55,213
Yoshi	18%	80.90%	NC_042030.1	58,714

**Table 3:** Summary of unique proteins with the phage WIVsmall.

Protein	Strand	Location	AA Length	Best Match					
				Phage_Gene	Host	Phage Cluster	Predicted Function	Cov. %/ Ident. %	E Value
Gp58	-	30,830– 31,282	453	*TPA4_27*	*Tsukamurella paurometabola* CON55	Singleton	Hypothetical protein	100/74	7E-62
				*GMA1_27*	*Gordonia malaquae* G239	Singleton	Hypothetical protein	100/62	7E-46
				*REQ3_58*	*Rhodococcus equi* Requ28	Singleton	Hypothetical protein	100/58	2E-45
				*PatrickStar_38*	*Gordonia terrae* 3612	CX	Hypothetical protein	96/58	3E-43
				*Keelan_56*	*Gordonia terrae* 3612	DP	Hypothetical protein	100/55	2E-39
Gp59	-	31,299– 31,745	447	*IdentityCrisis_23*	*Mycobacterium smegmatis* mc^2^155	Singleton	Hypothetical protein	32/56	7E-08
				*Shweta_22*	*Mycobacterium smegmatis* mc^2^155	N	Hypothetical protein	34/49	8E-07
				*Ruthiejr_30*	*Mycobacterium smegmatis* mc^2^155	K4	Hypothetical protein	34/49	8E-07
				*Willsammy_23*	*Mycobacterium smegmatis* mc^2^155	P1	Hypothetical protein	34/46	7E-06
				*Taquito_28*	*Mycobacterium smegmatis* mc^2^155	K4	Hypothetical protein	34/46	7E-06
Gp61	-	32,291– 33,682	1392	*Moosehead_28*	*Gordonia terrae* 3612	CZ6	Hypothetical protein	67/25	7E-07
				*Wilder_24*	*Mycobacterium smegmatis* mc^2^155	L2	Minor tail protein	40/23	1E-06
				*MkaliMitinis3_24*	*Mycobacterium smegmatis* mc^2^155	L2	Minor tail protein	50/23	1E-06
				*LilDestine_24*	*Mycobacterium smegmatis* mc^2^155	L2	Minor tail protein	50/23	1E-06
				*Lewan_24*	*Mycobacterium smegmatis* mc^2^155	Singleton	Minor tail protein	50/23	1E-06

**Table 4:** Mass spectrometry analysis of the mycobacteriophage WIVsmall.

No.	Detected Proteins	Predicated Function	Molecular Mass
1	Gp60	Phage minor tail protein	59.30 kDa
2	Gp62	Phage minor tail protein	63.75 kDa
3	Gp64	Phage minor tail protein	64.63 kDa
4	Gp65	Putative structural protein	63.52 kDa
5	Gp67	Tail-length tape measure protein	122.46 kDa
6	Gp73	Major tail protein	29.82 kDa
7	Gp77	Major capsid subunit	31.73 kDa
8	Gp80	Putative portal protein	51.45 kDa

## Data Availability

The GenBank accession number for the phage WIVsmall is KC736071.1.
